# The patient reporting and action for a safe environment (PRASE) intervention: a feasibility study

**DOI:** 10.1186/s12913-016-1919-z

**Published:** 2016-11-28

**Authors:** Jane K. O’Hara, Rebecca J. Lawton, Gerry Armitage, Laura Sheard, Claire Marsh, Kim Cocks, Rosie R. C. McEachan, Caroline Reynolds, Ian Watt, John Wright

**Affiliations:** 1 Yorkshire Quality and Safety Research Group, Bradford Institute for Health Research, Bradford Teaching Hospitals NHS Foundation Trust, Duckworth Lane, Bradford, BD9 6RJ England; 2 Leeds Institute of Medical Education, University of Leeds, Level 7 Worsley Building, Clarendon Way, Leeds, LS2 9NL England; 3 School of Psychology, University of Leeds, Lifton Place, Leeds, LS2 9JZ England; 4 School of Health Studies, University of Bradford, Richmond Road, Bradford, BD7 1DP England; 5 York Trials Unit, Department of Health Sciences, University of York, ARRC Building, York, YO10 5DD England; 6 Department of Health Sciences/Hull York Medical School, Faculty of Science, University of York, Area 4, Seebohm Rowntree Building, Heslington, York, YO10 5DD England

## Abstract

**Background:**

There is growing interest in the role of patients in improving patient safety. One such role is providing feedback on the safety of their care. Here we describe the development and feasibility testing of an intervention that collects patient feedback on patient safety, brings together staff to consider this feedback and to plan improvement strategies. We address two research questions: i) to explore the feasibility of the process of systematically collecting feedback from patients about the safety of care as part of the PRASE intervention; and, ii) to explore the feasibility and acceptability of the PRASE intervention for staff, and to understand more about how staff use the patient feedback for service improvement.

**Method:**

We conducted a feasibility study using a wait-list controlled design across six wards within an acute teaching hospital. Intervention wards were asked to participate in two cycles of the PRASE (Patient Reporting & Action for a Safe Environment) intervention across a six-month period. Participants were patients on participating wards. To explore the acceptability of the intervention for staff, observations of action planning meetings, interviews with a lead person for the intervention on each ward and recorded researcher reflections were analysed thematically and synthesised.

**Results:**

Recruitment of patients using computer tablets at their bedside was straightforward, with the majority of patients willing and able to provide feedback. Randomisation of the intervention was acceptable to staff, with no evidence of differential response rates between intervention and control groups. In general, ward staff were positive about the use of patient feedback for service improvement and were able to use the feedback as a basis for action planning, although engagement with the process was variable. Gathering a multidisciplinary team together for action planning was found to be challenging, and implementing action plans was sometimes hindered by the need to co-ordinate action across multiple services.

**Discussion:**

The PRASE intervention was found to be acceptable to staff and patients. However, before proceeding to a full cluster randomised controlled trial, the intervention requires adaptation to account for the difficulties in implementing action plans within three months, the need for a facilitator to support the action planning meetings, and the provision of training and senior management support for participating ward teams.

**Conclusions:**

The PRASE intervention represents a promising method for the systematic collection of patient feedback about the safety of hospital care.

**Electronic supplementary material:**

The online version of this article (doi:10.1186/s12913-016-1919-z) contains supplementary material, which is available to authorized users.

## Background

The perspective of patients has never been a more central concern to the way healthcare is delivered. Within the UK, a series of recent reports all emphasized the need to elicit, understand and respond to patient feedback about care [1–3]. This focus has been mirrored internationally, with ‘Partnering with Consumers’ in Australia,[4] and ‘Better Together’ in the US [5].

Traditionally, the way health services asked for patient feedback was to focus on patient satisfaction and experience, but over the past decade, researchers and practitioners alike have begun to understand how patients may also provide useful information to healthcare organisations about the safety of care [6–10]. Evidence is building regarding the ability and willingness of patients across healthcare settings to report on safety issues [10, 11]. However, evidence is lacking regarding concrete interventions allowing staff to use patient feedback about safety to improve safety performance. That is, we can evidently measure patient concerns about specific safety issues but what has yet to be established is how or if these may be used to improve safety, or indeed services more widely.

Additionally, most empirical work has focused on patient reporting of specific safety concerns or incidents, [12] which can be conceptualized in terms of human error theory [13] as ‘retrospective indicators’ of safety. What has not been considered is whether patients are also in a position to report on factors that contribute to future error – ‘prospective indicators’ of patient safety. Recent work has aimed to address this, with the development of the Patient Measure of Safety (PMOS) [6, 7] that allows the systematic collection of patient feedback about contributory factors to safety incidents, thereby moving us beyond simply measuring past ‘harm’ - a recent criticism of the way safety is currently measured [12].

This paper describes the development and feasibility testing of the PRASE (Patient Reporting and Action for a Safe Environment) intervention. This intervention represents an innovative approach for acute healthcare settings that puts patient feedback about safety, central to service improvement. The intervention uses two tools to ask patients about their experience of care within a hospital setting. These tools – which have been developed with patients and staff and published previously, [6, 7, 14] – sample both retrospective indicators (patient reported safety concerns) and prospective indicators (patient feedback about contributory factors to future adverse events) of patient safety. This information is then fed back to healthcare professionals on hospital wards, to be considered within an action planning process, with the aim of improving patient safety. The intervention is based on the principles of action planning, [15] with an articulated and systematic process of data feedback, problem solving, identification of actions and then monitoring of implemented actions. This approach, where measurement is regarded not as an end in itself, but rather a starting point for action and patient safety improvement, has been a key facet of other patient safety interventions, [16] and was designed to support the progression from measurement through to action and change.

The revised Medical Research Council Guidance for complex interventions, [17] outlines a systematic approach for their development and evaluation, with an emphasis on feasibility testing and piloting. The PRASE intervention clearly meets the definition of a complex intervention, with four interacting components, and a variety of behaviours required across a number of groups and organizational levels, resulting in localized tailoring of the intervention and the outcome measures used [17]. This feasibility study allows testing of intervention procedures (detailed within methods section below), exploring acceptability of the trial design, and estimating recruitment and retention, prior to testing within a wider efficacy cluster randomized controlled trial (cRCT) [18].

Specifically therefore, we had two key research objectives:

1. to explore the feasibility of the process of systematically collecting feedback from patients about the safety of care as part of the PRASE intervention;
2. to explore the feasibility and acceptability of the PRASE intervention for staff, and to understand more about how staff use the patient feedback for service improvement;

By addressing these, we aimed to identify refinements to the PRASE intervention, processes for collection of associated measures, and development of a logic model to support a wider cRCT.

## Methods

### Developing the PRASE intervention

The PRASE intervention is a complex multi-faceted intervention. Patient feedback about the safety of care is gathered using two measurement tools: the Patient Measure of Safety (PMOS), and Patient Incident Reporting Tool (PIRT). The development of these two tools has been described previously [6, 7, 14, 19]. However, in addition to the measurement of patient feedback, this intervention represents a practical process for receiving and acting upon patient feedback about safety, and as such needed further development. To develop this broader intervention we formed an Intervention Development Group (IDG). In addition to the research team this group comprised: three medical consultants and a consultant surgeon, an assistant chief nurse, a matron, three patient representatives, a lead for patient safety, a lead for patient experience, and a health economist. This group met four times over four months between January and May 2012, with the aim of identifying and explicating how patient feedback about safety might be used to improve ward-level patient safety, and how this would be implemented within acute NHS Trusts.

**Fig. 1:**
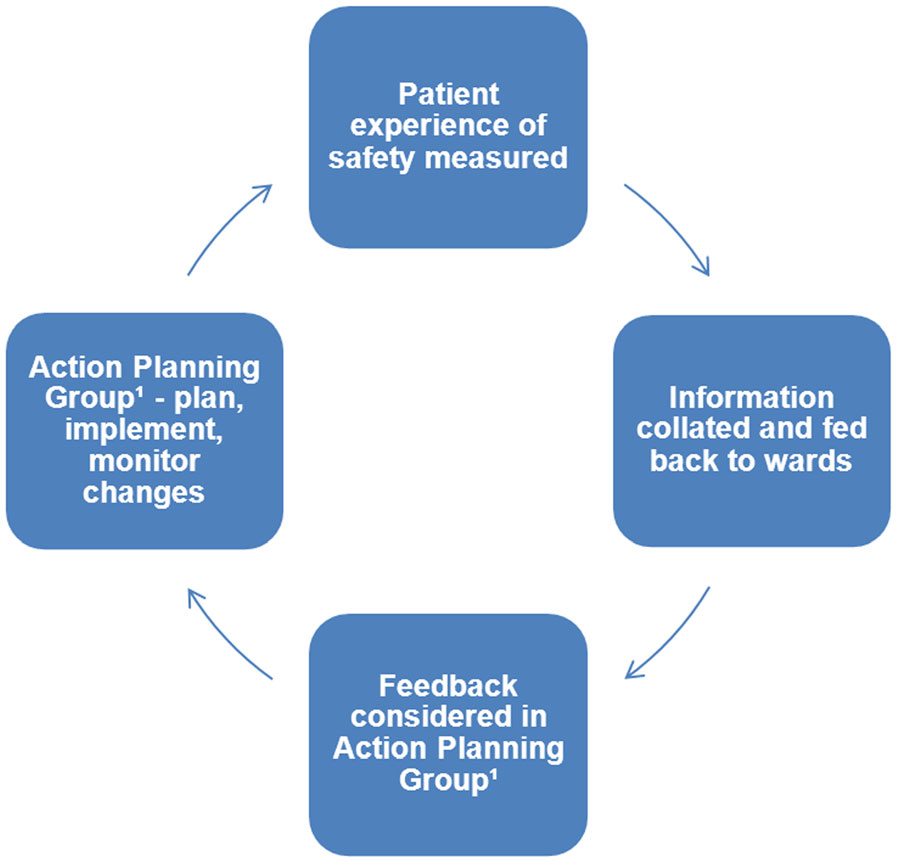
The PRASE Intervention cycle. The Action Planning Group comprised staff from the ward/unit from a range of professional backgrounds. A member of the research team observed the meeting, which was led by a member of the group itself

Figure 1 provides an illustrative summary of the PRASE intervention. The intervention commences with the measurement of the patient experience of safety using the two tools described previously. This information is then collated and fed back to ward staff in the form of a feedback report, the style and content of which was developed as part of the IDG. The feedback report is then discussed in a multi-disciplinary action planning group (APG). Within the IDG it was felt that the membership of the APG needed to reflect the different professions that deliver care on a ward, to fully understand the nature of the patient feedback, as well as to ensure a multi-disciplinary approach to agreeing, implementing and monitoring actions. The last key facet of the intervention is that it is a cycle, where the process of measurement, feedback and action planning is repeated. The prevailing view of the members of the IDG was that the cycle could be achieved within 3 months, and this therefore formed the basis of this feasibility study design.

### Testing the PRASE intervention: a feasibility trial

#### Setting and participants

This study was undertaken in a large acute teaching hospital in the North of England. Six wards were purposively sampled to represent a wide range of patient demographics, clinical specialty, and admission type (acute/elective). Specialties included were: i) paediatric surgery; ii) Ear Nose and Throat surgery; iii) medical admissions; iv) orthopaedics; v) renal, gastroenterology and rheumatology; and vi) urology. Participants in the study were patients on participating wards. Staff on participating wards were part of this study, but not as consented participants.

#### Design

The study used a wait-list cRCT design (see Additional file 1 for diagram). This design was used to test the feasibility of randomizing wards (including impact on recruitment of participants), and acceptability of the randomization allocation. Randomization was undertaken following the purposive sampling of the wards. In order to focus on the intervention fidelity this randomization was in a 2:1 ratio of intervention to control wards. Outcomes were measured at three points (baseline, three months, six months). Intervention wards received the PRASE intervention, with two 3-month cycles of patient feedback and action planning. Patient feedback on control wards was also collected at three time points, but these data were not fed back to staff until the end of the study. Data were collected between July 2012 and March 2013. Wards were randomly assigned to condition using a random number generator. Patient participants were blinded to randomisation, but research staff collecting the feedback, and ward staff interpreting and acting on this feedback, were not blinded.

### Procedure

#### Conducting the PRASE intervention

i) Collecting patient feedback about the safety of their care
  Patients were recruited in a staggered series of two week blocks across each six-week data collection period. Recruitment proceeded within two wards in the first two weeks, followed by another two wards in weeks 3-4, and the final two wards in weeks 5-6. The process of recruiting a patient started by asking staff on the ward to identify those patients that were unable to participate. Exclusion criteria included being too ill (excluding paediatrics, where relatives were able to take part on behalf of the patient), or not having capacity to consent (as judged by a senior nurse on the ward). A researcher then approached those patients who were eligible to participate and asked if they would be willing to complete a brief questionnaire. For all participants, written consent was obtained before proceeding further. On the paediatric ward within the sample, parents provided proxy consent for the participant (if aged under 16), with the questionnaire usually completed by the parents or guardians, although in some cases this was with input from the child or young adult. Once consented, the patients’ demographic information was recorded, and the PRASE measurement tools were completed (Additional file 2). All data were captured on computer tablets at the patient’s bedside, using software developed by the research team.^1^ This software allowed the direct input of data, and secure transfer to a database using an internal Wi-Fi connection within the hospital. Although participants could self-complete, the default for collection of these data was through a facilitated discussion with a researcher, as this has been found to be the best means of collecting this type of information from patients [14, 20].
  Where completion of the questionnaire was facilitated, the researcher would go through each of the questions on the PMOS in turn, noting the participants preferred response. Where patients volunteered other information to provide context for their answers, this could be recorded in three ways: i) as a general comment; ii) as a positive experience of care; or iii) as a ‘safety concern or experience’ through the PIRT. The decision as to how this additional information was recorded was decided upon within a discussion between the researcher and the patient, with the patient always having the final decision. Where a participant identified a ‘safety concern or experience’, the researcher would record this using a series of prompts, asking patients to consider what happened, why it was a safety concern for them, what could be done to prevent it happening again, as well as their perspective on the preventability and severity of the experience. The full list of PMOS questions and the PIRT proforma is provided in Additional file 2.
  Researchers aimed to collect 20-30 responses per ward over two weeks. It was anticipated that this would provide a robust representation of the patient experience of safety on the ward, be feasible within the two week timeframe, and provide an adequate amount of feedback for action planning.

ii) PRASE feedback report
  All feedback was collated and fed back to ward staff. Wards in the intervention received patient feedback at three time points: after baseline, after three months, and after six months. Wards in the control group were provided with feedback from all three time points at the end of the study. An example feedback report is provided in Additional file 3. A traffic light system was used to allow staff to quickly identify positive and negative responses, with safety concerns, comments and positive experiences presented alongside the relevant PMOS domains [7].

iii) Action planning and implementing change
  Intervention wards were asked to create a multi-disciplinary Action Planning Group (APG), within which the feedback could be considered and action plans made. Wards were advised to bring together staff from different professional groups and grades, in order to promote a more rounded interpretation of the patient feedback, and a shared approach to planning and implementing actions. The APG nominated someone from the group to write up and take responsibility for the action plan, using a standardized proforma supplied by the research team. This proforma asked staff to outline: i) a description of the identified problem; ii) action(s) identified; iii) who was responsible for leading on the actions identified; iv) the deadline for completing actions; v) how the team would measure success; vi) what the progress was against the action plan at a later review; and, vii) the completion date. The APG was asked to provide researchers with a copy of the completed proforma, to evidence the outcomes of the meeting, and allow enquiry about implementation of actions at the end of the study. Each APG meeting was attended by a researcher to observe the action planning process.

### Exploring the feasibility of the PRASE intervention

#### Research aim 1: To explore the feasibility of the process of systematically collecting feedback from patients about the safety of care as part of the PRASE intervention

The focus of this feasibility study was not on formal comparisons between intervention and control groups. Rather, we were concerned with i) exploring the feasibility of recruitment of participants and the impact of randomization of wards; and ii) examining the data for variation across wards and timepoints. This would inform the design of a subsequent larger randomized trial.

### Analysis

To examine the recruitment of patients, participant demographics were calculated across wards, and confidence intervals for the response rates calculated across intervention and control wards. To examine variation in the data generated by the PRASE measurement tools, an overall mean PMOS score, together with nine domain scores were calculated using the mean of two or more responses. Summary scores were calculated for the item ‘Were you treated with dignity and respect?’, which was regarded as a stand-alone item. In addition, the potential impact of method of completion (self-completion versus facilitated completion) was explored by examining the mean scores at baseline, and 6 months, for the overall PMOS score across all wards. Patient reported safety concerns were examined by calculating for each ward: i) the total number of concerns; ii) the number of patients reporting one or more concerns; iii) the mean number of reports per participant; iv) the mean (patient assessed) severity for reported concerns; v) the range of the (patient assessed) severity of reported safety concerns; and, vi) the average level of patient assessed preventability of reported safety concerns (expressed as the median due to it being an ordinal variable).

#### Research aim 2: To explore the feasibility and acceptability of the PRASE intervention for staff, and to understand more about how staff use the patient feedback for service improvement

Our second aim was to explore the feasibility and acceptability of the PRASE intervention as a basis for service improvement, and to understand more about how staff use the patient feedback. The focus here was on staff attitudes towards the intervention, as previous studies developing the measurement tools had focused more exclusively on the acceptability and feasibility for patients [6, 7, 14]. To explore the feasibility and acceptability of the PRASE intervention for staff, three qualitative methods were used. First, research staff made structured observational notes about each APG meeting they had attended. Second, follow up telephone interviews with the appointed PRASE lead for each of the intervention wards were conducted. Lastly, members of the research team recorded their impressions and tacit knowledge about the study via documented fortnightly team meetings. The observation guide for the APG meetings and the topic guide for the interviews are presented in Additional file 4.

### Analysis

Data from the three qualitative methods used (observational notes, phone interviews and recording of tacit knowledge) were each subjected to a thematic analysis [21] and then synthesized. Our coding framework related to directly addressing research aim 2, (exploring the acceptability and feasibility of the PRASE intervention). Therefore, coding specifically pertained to issues of acceptability and feasibility. The combined results from the quantitative and qualitative analysis described above were then triangulated, and considered within the research team with the aim of directing refinements to the PRASE intervention and to specify precise details of the design of the wider cRCT.

This study was approved by a National Health Service research ethics committee.

## Results

### Research Aim 1: To explore the feasibility of the process of systematically collecting feedback from patients about the safety of care as part of the PRASE intervention

#### Feasibility of patient recruitment

A total of 379 patients were recruited into the study over the six-month study period. Table 1 shows participant characteristics by ward. The average number of participants recruited per ward at each time point was 20.9, with a mean response rate of 86.8%. Reasons for refusal included patients feeling too unwell, feeling that they had no substantive feedback to give, or lack of time due to an imminent test or procedure. Length of stay at point of consent ranged between 1 and 9.7 days, with an overall mean of 3.4 days. The primary ethnic origin of participants was “White British” (79%), with the overall ethnic group representation across the study approaching that of the local population [22].

**Table 1:** Patient participant characteristics by ward

Ward	Patient participants consented (response rate %)			Mean age (years)	Gender	Ethnicity	Mean length of stay at consent (days)	Self-completion of questionnaire (% of participants recruited)		
	Baseline	3 months	6 months					Baseline	3 months	6 months
Ward A^a^ *(Control)*	20 (95)	24 (100)	21 (100)	8	38 Male	49% White British	1	9 (45)	11 (46)	12 (57)
					25 Female	41% Pakistani				
						10% Other				
Ward B *(Intervention)*	26 (100)	21 (88)	20 (80)	50	35 Male	85% White British	1.3	6 (23)	4 (19)	4 (20)
					32 Female	9% Pakistani				
						6% Other				
Ward C *(Intervention)*	21 (95)	21 (70)	20 (80)	52	30 Male	85% White British	9.7	2 (10)	1 (5)	6 (30)
					32 Female	8% Pakistani				
						7% Other				
Ward D *(Intervention)*	22 (96)	20 (67)	20 (95)	58	42 Male	79% White British	4.3	6 (28)	5 (25)	6 (30)
					20 Female	13% Pakistani				
						8% Other				
Ward E *(Control)*	20 (77)	20 (74)	20 (87)	48	34 Male	83% White British	2.5	6 (30)	5 (25)	8 (40)
					26 Female	12% Pakistani				
						5% Other				
Ward F *(Intervention)*	21 (100)	19 (79)	20 (80)	59	29 Male	92% White British	7.3	5 (24)	3 (16)	4 (20)
					31 Female	5% Pakistani				
						3% Other				
Summary – Control	40 (95.24; CI 88.8-100.0)^b^	44 (81.4; CI 71.1-91.8)^b^	41 (97.6; CI 93.0-100.0)^b^	28	72 Male	66% White British	1.75	15 (38)	16 (36)	20 (49)
					51 Female	27% Pakistani				
						7% Other				
Summary - Intervention	90 (97.83; CI 95.0 -100.0)^b^	81 (77.1; CI 69.1-85.1)^b^	80 (81.6; CI 74.0-89.3)^b^	55	136 Male	85% White British	5.65	19 (21)	13 (16)	20 (25)
					115 Female	9% Pakistani				
						6% Other				
Summary – Overall	130 (93.5; CI 89.4-97.6)^b^	125 (78.6; CI 72.2-85.0)^b^	121 (86.4; CI 80.8-92.1)^b^	46	208 Male	79% White British	4.35	34 (26)	29 (23)	40 (33)
					166 Female	15% Pakistani				
						6% Other				

^a^In this ward, the participant was the child or young adult receiving care, but proxy consent was taken from the parents

^b^95% confidence intervals (CI) for response rates are provided alongside the percentage response rate

These participant characteristics demonstrate that we were able to collect feedback from a variety of patients, across a range of hospital settings. We recruited both males and females, patients of varying age, and from different ethnic groups. Of interest is the differing length of inpatient stay at time of consent. Whilst this reflects the patient turnover across different wards, it also highlights the willingness of patients to report on their experience of safety after a very short period of time in hospital. Lastly, the response rates reflect the acceptability of the PRASE intervention for patients, with the majority of patients approached agreeing to participate in the study.

With the exception of one ward at one timepoint (an orthopaedics ward, with a higher average patient age during the winter period), we found that we were able to achieve our aim of collecting data from 20-30 patients on all wards, within a two week period. As can be seen from the PMOS and patient reported safety concerns data, this target figure yielded a variation in scores, and a range of qualitative data from the safety concerns.

### Exploring impact of randomisation

As can be seen in Table 1, the 95% confidence intervals for summary response rates (intervention and control) indicate that the consent rate was slightly higher in the control group compared to the intervention. This is likely because one of the control wards was a paediatric ward, where those consented were parents (providing proxy consent on behalf of their child), and therefore not unwell, as well as motivated to feedback their experience of care. Overall however, the patient consent rate was very good, and did not drop significantly over time. All wards were also retained in the study. This indicates that the process of randomisation was acceptable to both control and intervention wards, with no indication that response rates might differ between groups.

### Exploring variation within the PRASE measurement tools

Table 2 displays the means and standard deviations for each of the PMOS domains and the patient reported safety concerns, by ward, at baseline and six months. Data are not presented for the second time point due to the fact that some action planning groups were not able to meet within the first three month cycle. This is discussed in more detail in the next section.

**Table 2:** Ward summaries: PMOS domain scores and patient reported safety concerns at baseline, and at 6 months

	Ward A *(Control)*		Ward B *(Intervention)* Mean (SD)		Ward C *(Intervention)* Mean (SD)		Ward D *(Intervention)* Mean (SD)		Ward E *(Control)* Mean (SD)		Ward F *(Intervention)* Mean (SD)		Summary – Control Mean (SD)^c^		Summary – Intervention Mean (SD)^c^	
	Baseline	6 months	Baseline	6 months	Baseline	6 months	Baseline	6 months	Baseline	6 months	Baseline	6 months	Baseline	6 months	Baseline	6 months
Dignity & respect^a^ Mean (SD)	4.47 (0.61)	4.19 (0.93)	4.54 (0.76)	3.75 (1.37)	4.19 (0.68)	4.30 (0.47)	4.45 (1.05)	4.38 (0.61)	4.25 (0.71)	4.45 (0.60)	4.00 (1.09)	4.10 (1.02)	4.36 (0.16)	4.32 (0.18)	4.29 (0.25)	4.14 (0.29)
Access to resources^b^ Mean (SD)	3.85 (0.60)	4.16 (0.31)	3.93 (0.55)	3.94 (0.34)	3.80 (0.40)	3.55 (0.40)	4.08 (0.49)	3.86 (0.44)	4.00 (0.24)	3.69 (0.38)	4.07 (0.52)	3.89 (0.32)	3.93 (0.11)	3.91 (0.35)	3.97 (0.13)	3.81 (0.18)
Communication & teamworking^b^ Mean (SD)	4.07 (0.50)	4.18 (0.40)	4.10 (0.40)	3.96 (0.58)	3.92 (0.48)	3.99 (0.43)	4.10 (0.57)	4.16 (0.31)	4.14 (0.25)	3.94 (0.48)	4.02 (0.65)	3.94 (0.44)	4.10 (0.05)	4.06 (0.17)	4.03 (0.08)	4.01 (0.10)
Delays^b^ Mean (SD)	3.29 (1.16)	4.18 (0.46)	3.83 (0.69)	3.14 (0.99)	2.89 (0.91)	3.44 (0.64)	4.03 (0.62)	3.97 (0.76)	3.73 (0.78)	3.75 (0.79)	3.53 (0.90)	3.36 (0.95)	3.51 (0.31)	3.94 (0.33)	3.57 (0.49)	3.49 (0.36)
Equipment^b^ Mean (SD)	4.24 (0.50)	4.21 (0.40)	3.82 (0.64)	3.84 (0.35)	3.83 (0.47)	3.91 (0.32)	4.10 (0.74)	4.10 (0.39)	4.03 (0.34)	4.03 (0.40)	4.19 (0.68)	3.97 (0.58)	4.13 (0.15)	4.11 (0.13)	3.98 (0.19)	3.96 (0.11)
Information Flow^b^ Mean (SD)	3.71 (0.74)	3.95 (0.57)	3.87 (0.62)	3.84 (0.64)	3.76 (0.42)	3.61 (0.86)	3.89 (0.54)	3.82 (0.49)	3.78 (0.85)	3.68 (0.60)	3.75 (0.65)	3.74 (0.57)	3.75 (0.47)	3.81 (0.20)	3.82 (0.07)	3.75 (0.10)
Organisation & care planning^b^ Mean (SD)	3.94 (0.63)	4.16 (0.43)	4.00 (0.54)	3.83 (0.57)	3.86 (0.54)	3.98 (0.50)	4.06 (0.65)	3.97 (0.44)	4.04 (0.57)	3.86 (0.38)	3.84 (0.63)	3.85 (0.63)	3.99 (0.07)	3.99 (0.24)	3.94 (0.11)	3.91 (0.09)
Staff roles & responsibilities^b^ Mean (SD)	3.39 (0.90)	3.65 (0.77)	3.27 (0.90)	3.54 (0.74)	3.52 (0.77)	3.42 (0.88)	3.56 (0.96)	3.39 (0.76)	3.85 (0.60)	3.11 (1.07)	3.27 (0.95)	3.32 (0.91)	3.62 (0.32)	3.40 (0.35)	3.41 (0.16)	3.41 (0.09)
Staff training^b^ Mean (SD)	3.75 (1.05)	4.23 (0.39)	3.63 (0.85)	3.90 (0.43)	3.87 (0.33)	3.69 (0.84)	3.97 (0.89)	3.97 (0.28)	4.08 (0.46)	3.83 (0.79)	4.03 (0.72)	4.03 (0.13)	3.92 (0.24)	4.06 (0.24)	3.87 (0.18)	3.88 (0.14)
Ward type & layout^2^ Mean (SD)	3.68 (0.70)	3.86 (0.64)	3.70 (0.59)	3.48 (0.60)	3.49 (0.37)	3.67 (0.46)	4.03 (0.59)	3.77 (0.51)	4.00 (0.48)	3.82 (0.52)	3.79 (0.55)	3.81 (0.26)	3.84 (0.23)	3.85 (0.03)	3.76 (0.22)	3.68 (0.14)
PMOS overall score^b^ Mean (SD)	3.73 (0.59)	4.18 (0.36)	3.72 (0.32)	3.70 (0.39)	3.65 (0.38)	3.70 (0.27)	4.05 (0.39)	3.89 (0.26)	3.90 (0.28)	3.73 (0.36)	3.80 (0.51)	3.82 (0.32)	3.81 (0.12)	3.95 (0.32)	3.80 (0.18)	3.78 (0.09)
No. of patient reported safety concerns	6	1	7	17	6	35	16	8	8	9	15	19	7	5	11	20
No. of patients reporting one or more concerns	3	1	3	8	4	12	6	7	5	5	8	8	4	3	6	9
No. of reports per patient recruited	0.3	0.05	0.27	0.85	0.29	1.75	0.73	0.4	0.4	0.45	0.71	0.95	0.35	0.25	0.5	0.93
Average severity	1.5	4	9	8	7	7	7	5	6	7	6	6	3.75	5.5	7.5	6.5
Range of severity	1-2	-	8-10	5-10	5-10	2-10	1-10	2-8	2-8	3-9	3-10	2-9	-	-	-	-
Average preventability^d^	Definitely yes, definitely no	Probably yes	Definitely yes	Probably yes	Definitely yes	Probably yes	Definitely yes	Probably not	Definitely yes	Probably yes	Probably yes	Probably not	-	-	-	-

^a^Dignity & respect is a one item measure, and is not included within a PMOS domain, nor within the overall PMOS score. It is used for interpretive purposes for ward staff during the action planning process

^b^Figures represent mean scores, calculated on the basis of two or more responses per domain (where the domain is more than two items). The PMOS overall score represents the mean score across all nine domains. SD refers to standard deviation

^c^This figure represents a cluster-level mean

^d^Due to the ordinal nature of this variable, the average is presented using the median, and no summary cluster-level averages are presented for the intervention and control wards

The PMOS domain scores show some variation across wards. For example, in the domain ‘delays’, scores range between 2.89 and 4.03 at baseline (higher scores represent more positive responses). There is also variation within wards, both across the domain scores and the two measurement points. For example, for some domains there is an increase in scores between baseline and six months (e.g. for ‘delays’ Ward C increased from 2.89 to 3.44), whereas for others there is a reduction (e.g. for ‘delays’ Ward F decreased from 3.53 to 3.36). The dignity and respect measure also shows similar patterns of variation.

The additional analysis to explore the impact of method of completion on PMOS scoring (self-completion or facilitated discussion), suggested that this made little difference. At baseline, the mean overall PMOS score for self-completion was 3.84 (SD = 0.57), and 3.79 (SD = 0.39) for facilitated completion. At 6 months, the mean overall PMOS score for self-completion was 3.81 (SD = 0.43), and 3.81 (SD = 0.32) for facilitated completion.

With respect to the patient reported safety concerns, the figures suggest that patients are able to feedback safety concerns as part of the PRASE tool. Across all wards and time points, the mean number of reported safety concerns per patient recruited ranged from 0.05 to 1.75, with variation in terms of their perceived severity and preventability. Of note is the increase in patient reports for Ward C between baseline (6 reports) and six months (35 reports). The exact reason for this is unclear. The change is not accounted for by practice or seasonal effects, as the number of reports is decreasing on other wards. On examining the content of these reports, no consistent themes emerged, rather just a greater volume of reported safety concerns by patients. It is possible that there were changes occurring in the ward that would provide some context for this change, which we have not picked up in the qualitative enquiry, being focused as it was on the feasibility of the intervention.

### Research Aim 2: To explore the feasibility and acceptability of the PRASE intervention for staff, and to understand more about how staff use the patient feedback for service improvement

Seven action planning meetings (out of a possible eight) were held across the two phases of the study. This consisted of three (out of a possible four) action planning meetings in phase one and four (out of a possible four) action planning meetings in phase two. Observational notes were taken for all intervention wards at both stages of the project (*n* = 6) apart from for one ward at stage one, due to an administrative error. Action planning meetings ranged from 20 to 60 minutes in length with the average length being 37 minutes. The number of staff in each action planning meeting ranged from one to five with the average being three. The majority of these meetings consisted of nursing staff despite the efforts of the research team to involve medical staff. One ward involved a consultant and another ward involved an occupational therapist and healthcare assistant. The meetings were held in offices near to the ward where the staff worked. Staff from three of the four intervention wards took part (*n* = 6) in the telephone interviews, with staff from the remaining ward declining due to lack of availability. From a synthesis of the qualitative methods (observational notes, telephone interviews and meeting minutes), three themes arose, which are useful to examine in depth and are detailed under the headings below.

### Staff attitudes towards the intervention

The attitude of ward staff towards the intervention was a recurrent theme within our data, with consistency observed across the different qualitative methods. Overall, staff from three wards were largely positive about the PRASE process, welcoming the opportunity to receive detailed patient feedback and facilitated action planning. They valued the patient feedback itself as well as the process of involving a multi-disciplinary team in addressing the concerns that their patients had raised. An interesting point to note – that arose through all methods – is that when staff viewed the intervention positively, they often talked about how data from this study had reinforced data they were receiving through other safety initiatives or even what was known tacitly about the ward. It seems that patient feedback collected and presented through the PRASE intervention added a level of external validity to issues staff had previously not felt able to progress, because it was systematically collected from an independent research team.

However, staff acceptance across different wards did vary. On one of the four wards staff representatives were largely indifferent to the patient feedback contained in their PRASE report and chose not to make action plans in either phase of the project. They stated that most of the issues that patients raised in the feedback report would already be addressed on a daily basis through their ongoing attention to patients’ needs.

It is important to recognise that attitudes towards the intervention were not related to whether the feedback received from patients was on the whole largely positive or largely negative. One of the wards who were most welcoming of the intervention had received a significant number of negative responses from patients and they were keen to learn from these. By contrast, the ward with staff most dismissive of the intervention had some of the most positive patient feedback. Therefore it cannot be concluded that a ward with poorer patient feedback have staff who are too busy or ‘stressed’ to be able to engage with the intervention. Instead, other factors such as the extent to which they view the intervention as a vehicle to support continuous improvement through learning, are likely to be important. This level of acceptance is important – the ward that did not see the value in PRASE feedback, did not create action plans.

### Implementation of action plans

Action plans tended to consist of two or three main action points, although for some wards there were more. One ward chose not to make an action plan at either time point. Of the other three wards, 21 action points were made in total. The implementation of action plans was assessed through the telephone interviews, using a three point scoring system: ‘fully’, ‘partially’ or ‘not at all’. A score of ‘partially implemented’ was given to most of the action points made (57%), with 33% of action points fully implemented, and just 10% not implemented at all. Actions that were achieved were those that had a defined and relatively simple remit and could be accomplished by members of the APG. Examples include a review and alteration of furniture layout to provide more space around patient bedsides, and a new procedure for obtaining information about patients’ existing medications on arrival into hospital. This new procedure involved the provision of a medication box in the ward fridge so that patients could bring their medication from home – a simple but explicit change in admissions procedure that ensured full details of medications were available.

The two action points that were never achieved were dependent on engagement from members of staff in other departments. An example of this is an action for staff to contact porters about delays in transporting patients from the ward to elsewhere in the hospital, and to investigate delays at the time when they occurred. However, this approach did not resolve the issue because staff understanding of the porter system and how to influence its timeliness, limited their ability to influence change.

The majority of actions were partially implemented and we identify two main reasons that prevented full implementation. First, many actions were dependent on the speed and effectiveness of wider Trust initiatives. An example is the training of staff in the specialty-related needs of patients on the ward, which some patient feedback had revealed was not currently adequate. A Trust initiative to develop all junior nursing staff across a range of training indicators was being rolled out across the hospital at the time of the study but had not achieved completion by the time the study ended. Second, many actions were dependent on addressing staff habits that in some cases were felt to be too deeply entrenched to be open to modification. An example is for staff involved in a patient’s care to introduce themselves to that patient on a daily basis. Some staff responded well to this but others did not feel comfortable doing so.

The phone interview data revealed that ward staff need support from senior management in order to address patient feedback relating to issues that are beyond the direct control of a ward-based APG. One ward vocalized that the patient feedback about the ward needed to be directed to senior management in order for concrete change to be implemented. In addition, a longer time period was identified as being necessary in order to implement agreed actions, especially when they were dependent on wider Trust initiatives, or cultural change.

### The APG meeting

Although a time period of three months for holding an APG and progressing associated action plans, was recommended by the Intervention Development Group, in reality we found this timescale too short, with two out of four intervention wards failing to meet within the first three months. Wards were also largely unable to bring together an APG comprising different professions and grades, with only one out of eight APG meetings reflecting this aim. APG meetings were also largely held with more senior nursing staff, with three out of eight meetings held with the senior sister representing the ward on his/her own. Medical staff were under-represented in APG membership, attending only one of the eight meetings held.

Research staff initially attended APG meetings purely in an observational capacity. At the outset of the study, the ward staff in the APG were tasked with convening their own meeting, autonomously setting their own agenda and discussing the patient feedback amongst themselves. However, it quickly became apparent that researchers were being drawn into a quasi-facilitation role; otherwise the APG did not function as intended. In some APGs, research staff were asked for their opinion on the patient feedback, and even what action plans the researcher would want to make if they were a member of the ward team. As the study progressed, most researchers relinquished their purely observational role in order to ensure that action planning took place.

Within the research team, we held a reflective group discussion about the function of the facilitation role that researchers had found themselves in. The group of researchers agreed that facilitation of an APG was necessary for the following reasons, as it ensured: i) staff actually met in an APG to consider the feedback; ii) talked to each other about the patient feedback; iii) each member of the APG had an equal voice; iv) action plans were produced as a result of the APG meeting (as the facilitator encouraged this); and, v) the focus was kept on action plans based on the data from the study rather than changes the staff wanted to make, which were perhaps not patient-focused or identified.

## Discussion

This paper has described the process of developing and feasibility testing the PRASE intervention. The findings suggest that the process of systematically collecting feedback from patients on the safety of care can be achieved within the PRASE intervention. Our response rates illustrate that patients were very willing to provide feedback via the PRASE measurement tools, either through self-completing the questionnaire, or through a facilitated conversation at the bedside, the latter being the overwhelming preference for patients. Furthermore, we achieved these response rates across hospital settings (e.g. elective and acute surgery wards, and medical wards), across short and longer-term inpatient stays, and from a representative patient demographic. We were able to achieve our desired figure of 20-30 patients per ward across all but one ward at one measurement point. This figure has also been demonstrated to show some variability in scores and provide comprehensive and interpretable feedback reports for staff. Lastly, there was no evidence that randomisation caused any problems for recruitment of participants, or retention of wards within the study. These findings provide important information for the further testing of the PRASE intervention.

With respect to the feasibility and acceptability of the PRASE intervention for staff, our qualitative enquiry raised a number of important issues that affected the use and usefulness of the feedback for ward staff, and any resultant action on the basis of this feedback. First, the expectation that the APG could be scheduled in existing multi-disciplinary meetings on participating wards was unrealistic. The first challenge was to convene such a coming together of professionals. Given the very real service pressures currently experienced within the NHS, the second challenge was then to ensure that staff intending to participate could get time away from patient care to attend. This ultimately highlighted the need for the PRASE intervention to have explicit guidance regarding the investment in time required to support it, as well as a more realistic timeframe for the intervention ‘cycle’. Linked to this was our finding that wards struggled to undertake APG meetings that were multi-disciplinary and comprising different levels of staff. This narrowing of APG membership inevitably has a number of impacts, not least the over-representation of the nursing perspective in the interpretation of the feedback and subsequent actions. Additionally, the lack of more junior staff meant that sometimes the perspective of those delivering ‘frontline’ care was lacking, further compounding this issue. The almost complete lack of medical representation at the APG meetings raises the concern that the intervention may be regarded solely as a ‘nursing’ issue, due to its focus as a ward-level improvement intervention. Future iterations of the intervention should consider how to encourage and support other staff groups to be part of the action planning process.

Second, our findings suggest that there will be an inherent variation in the way that healthcare professionals perceive the role of patient feedback as a means of service improvement. Whilst we witnessed enthusiasm for this role, there was also some reticence and defensiveness, resulting in a lack of engagement with the process of action planning. Whilst some of this reticence was likely linked to service demands, our findings suggest that some of this will also reflect the attitude of staff. Whilst the former is outwith the control of an isolated intervention, the latter is something that future iterations of PRASE should address. One means of promoting the credibility of the patient perspective of care to support improvement, may be the inclusion of patient representatives within action planning meetings. Although this would likely present some challenges, not least the pragmatics of such an approach, it is certainly something that warrants further examination.

Third, despite the widespread view of healthcare professionals involved in helping develop the PRASE intervention - that action planning was a simple process that staff would not need support with - our findings suggested that this was not the case. Whilst we intended at the outset to simply observe the meetings and action planning, the reality was that observers were often drawn into a more facilitative role, with staff almost looking to them for guidance about how to interpret, synthesise, and act on patient feedback. In retrospect this is perhaps unsurprising, and it could be argued that assuming a ‘light-touch’ intervention would uniformly fit all levels of service improvement expertise was naïve. However, given that the level of support was guided by professionals within the Intervention Development Group, the discrepancy between perceived action planning competence and the reality, is of note.

Lastly, as has been highlighted in the recent high profile report on patient safety failings within a UK hospital, [1] action plans often result in little or no action. It is therefore important to consider how to support the implementation of actions within the PRASE intervention. Our findings suggest that actions falling within the scope of the ward team are more likely to be implemented successfully, whereas more systemic changes were less likely to be addressed within the study timeframe. A significant factor in the success of implementing actions was found to be the ability of staff to link with other units or support services, in order to make more systemic changes. Some actions, which arguably take a more ‘upstream’ systems approach, were stymied by the perceived helplessness of staff to affect change outside of the ward in which they are based. It would seem that to achieve change that has implications beyond individual wards, it will be necessary to engage the support of senior managers from the outset. Senior managers with a hospital-wide remit will bring with them access to information that may be relevant to desired actions (which may avoid duplication), potential resources (time, funding, influence), and a public commitment to using patient feedback to improve services. It will be necessary however, to manage the risk that increased senior management involvement leads to an erroneous perception of the intervention as a mechanism for ward-level performance management, and not a tool, as is intended, for local patient-centred service improvement.

### Recommended changes to PRASE intervention and cluster RCT design

In light of our findings from the feasibility testing, Table 3 outlines the recommended changes to the PRASE intervention process, prior to conducting the cRCT. It is recognised that such changes add to the resources required to conduct the PRASE intervention, and as such, health economic data will be collected as part of the cRCT, to conduct a cost-consequences analysis.

**Table 3:** Recommended changes to the PRASE Intervention

•	*an increase in time between cycles* of measurement, feedback and action planning, from three months to six months;
•	*facilitated action planning meetings* to encourage teams to integrate the different types of patient feedback, and support longer-term systems-based change over inappropriate ‘quick fixes’;
•	*an augmented action planning pro-forma* to encourage staff to consider how they will measure success in addition to what action they will undertake;
•	*better engagement with, and support from senior management* to support action planning and implementation process;
•	*initial training session* to introduce the intervention to staff, and describe more fully the process of using patient feedback for service improvement;
•	*update sessions* to provide an opportunity for shared learning between groups and with senior managers. Issues that transcend ward boundaries can be discussed and a wider improvement approach taken;
•	*to avoid duplication of effort* by explicitly recognising (within the APG facilitation guidance) the need for PRASE to fit within other service improvement activity.

### Developing a logic model

**Fig. 2:**
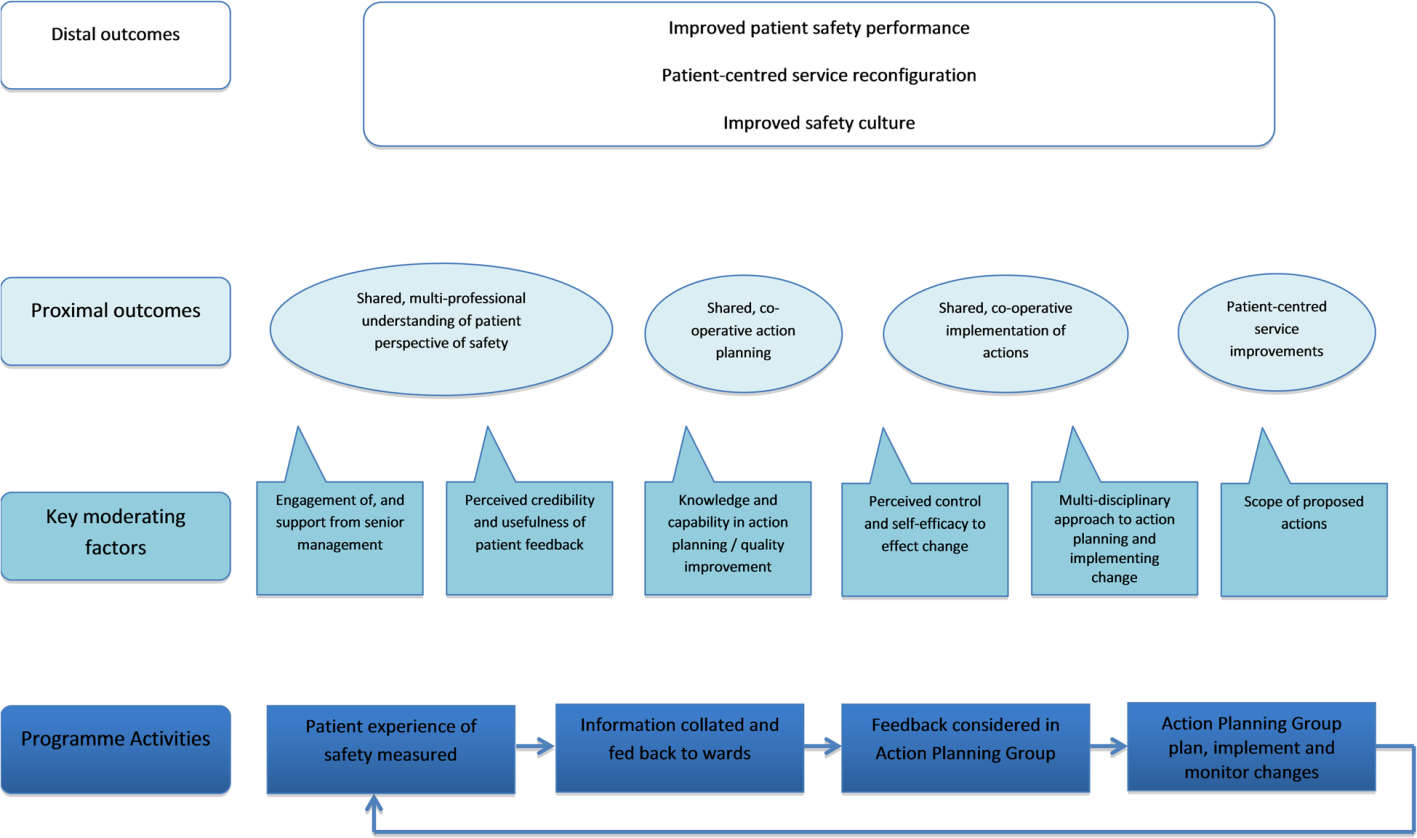
Outline logic model for the PRASE Intervention

Having an explicit theory of change for the implementation and evaluation of service improvement interventions, is becoming increasingly recognised as crucial for both researchers and improvers [23]. Figure 2 outlines the logic model based on the findings and recommendations of this study. This logic model will form the basis of the testing of the PRASE intervention in the subsequent cRCT [18].

### Limitations

This study had a number of limitations. First, whilst data from six wards was sufficient to address the feasibility study aims, it did not allow for estimates of effectiveness that may have informed the subsequent study further. Second, with respect to the recruitment of patients into the study, it is possible that in asking the nursing staff about the capacity of patients to participate, a bias is introduced into the sampling regimen. However, it is strongly felt by the research team that this risk must be balanced against the greater risk of causing distress to patients unable to participate due to acute illness or capacity to consent. Furthermore, given that engagement with the intervention by ward staff is an important factor, it was felt by the team that we must at all times work with staff in ways that were acceptable to them.

## Conclusion

The PRASE intervention represents an innovative approach to systematically collect feedback from patients about the safety of their care, as a basis for service improvement. It is possible that the intervention will be as much about changing culture as it will the specifics of service improvement. To this end, it will be important for health services seeking to use the intervention to provide adequate resources to support it, in terms of senior management support, as well as the time and space for staff to learn about, interpret and act on patient feedback about safety. It will be crucial not just to introduce this as a top-down safety intervention (the next ‘audit’ to achieve), focused as it is on encouraging ‘bottom-up’ change from the patient perspective upwards. If wards engage wholeheartedly with the intervention, it is possible that this will aid the journey towards achieving greater transparency in how we deliver care, as well as a public commitment to co-designing shared solutions to patient-centered problems.

## Additional files

Additional file 1: Outline study design. Diagram of study design. (PDF 174 kb)
Additional file 2: PRASE measurement tools (PMOS and PIRT). (PDF 179 kb)
Additional file 3: Example PRASE feedback report. (PDF 312 kb)
Additional file 4: Observation template for the Action Planning Group meetings. (PDF 75 kb)

## Abbreviations

APG: Action planning group
CI: Confidence interval
cRCT: Cluster randomised controlled trial
IDG: Intervention development group
PIRT: Patient incident reporting tool
PMOS: Patient measure of safety
PRASE: Patient reporting and action for a safe environment
SD: Standard deviation

## Footnotes

1 The software was developed by software developers employed at the Bradford Institute for Health Research, Bradford, UK
