# Supplementary figures and images for: The patient reporting and action for a safe environment (PRASE) intervention: a feasibility study
Source: BMC Health Serv Res. 2016 Nov 28;16:676. doi: 10.1186/s12913-016-1919-z (PMC5127050; doi:10.1186/s12913-016-1919-z)

## Appendix 1

### Outline study design

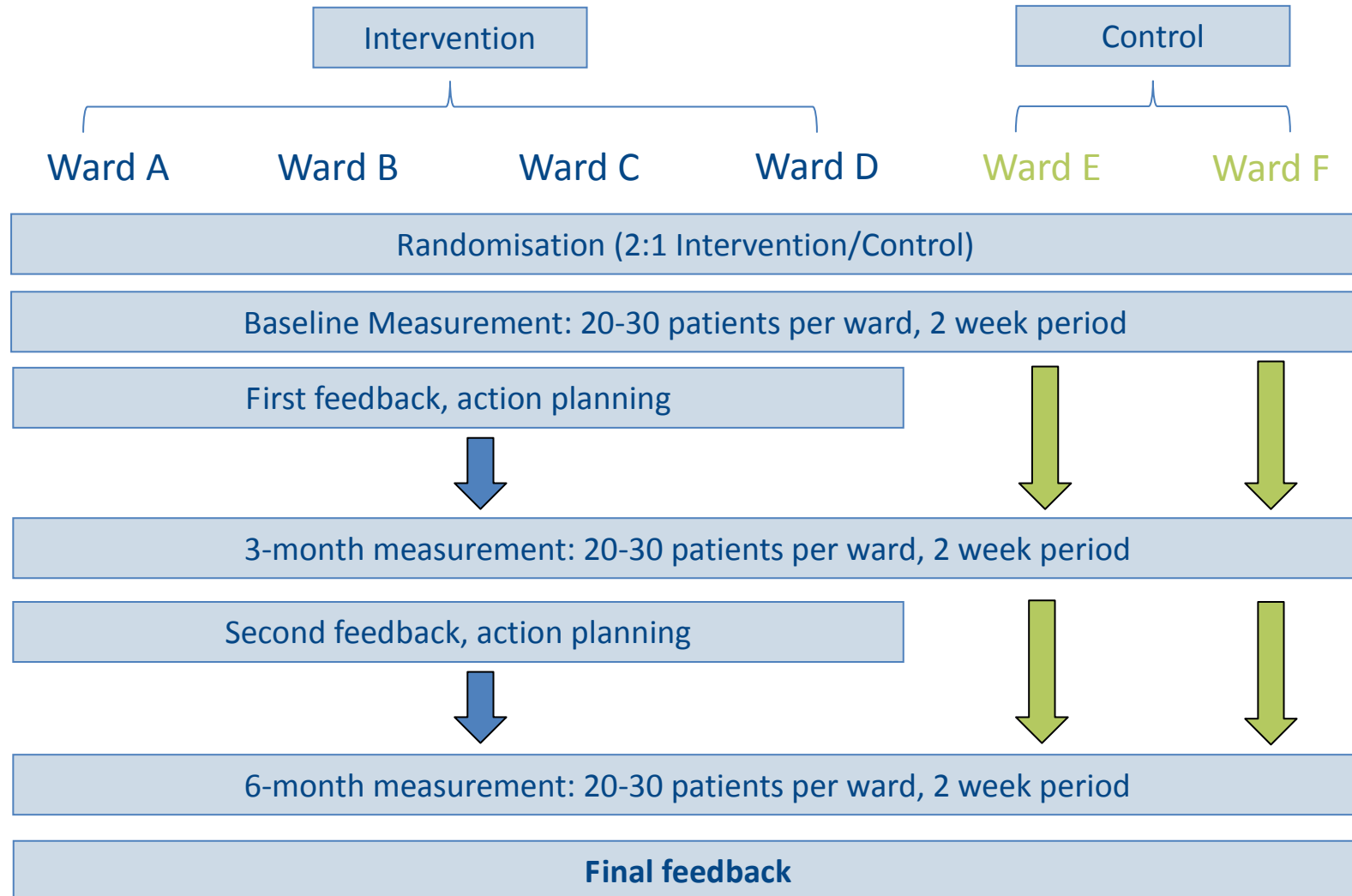

Supplement: Additional file 1: — Outline study design. Diagram of study design. (PDF 174 kb) [file 12913_2016_1919_MOESM1_ESM.pdf]
