# Supplementary material for: The patient reporting and action for a safe environment (PRASE) intervention: a feasibility study
Source: BMC Health Serv Res. 2016 Nov 28;16:676. doi: 10.1186/s12913-016-1919-z (PMC5127050; doi:10.1186/s12913-016-1919-z)
Supplement: Additional file 2: — PRASE measurement tools (PMOS and PIRT). (PDF 179 kb) [file 12913_2016_1919_MOESM2_ESM.pdf]

Appendix 2

Below are the two measurement tools that form the basis of the PRASE Intervention. These tools are represented in written format here, but please note that in the study all data were captured via software that allowed data entry directly onto a computer tablet at the patient's bedside.

i) PATIENT MEASURE OF SAFETY (PMOS)

	**Strongly Disagree**	**Disagree**	**Neither agree or disagree**	**Agree**	**Strongly Agree**	**Not Applicable**	**I prefer not to answer**	**Additional Comments**
1. I was always treated with dignity and respect	1	2	3	4	5	N/A		
2. I knew who to go to if I needed to ask a question	1	2	3	4	5	N/A		
3. The drugs I have been prescribed were always available	1	2	3	4	5	N/A		
4. I got answers to all the questions I had about my care	1	2	3	4	5	N/A		
5. Staff were always able to get advice from other teams about my care if needed	1	2	3	4	5	N/A		
6. A doctor changed my plan of care and other staff didn't know about it	1	2	3	4	5	N/A		
7. After a shift change staff did not appear to know important information about my care	1	2	3	4	5	N/A		

	Strongly Disagree	Disagree	Neither agree or disagree	Agree	Strongly Agree	Not Applicable	I prefer not to answer	Additional Comments
8. I knew what the different roles of the people caring for me were	1	2	3	4	5	N/A		
9. On at least one occasion a member of staff was not able to use the necessary equipment	1	2	3	4	5	N/A		
10. My treatment/ procedure/ operation did not always happen on time	1	2	3	4	5	N/A		
The following aspects of the ward made it difficult for **staff** to do their jobs:								
11. Position of nurses station	1	2	3	4	5	N/A		
12. Lighting levels	1	2	3	4	5	N/A		
13. Clutter & untidiness	1	2	3	4	5	N/A		
14. Lack of space	1	2	3	4	5	N/A		
15. I was on a ward that was not able to deal with my treatment needs	1	2	3	4	5	N/A		
16. Staff were prompt in answering my buzzer	1	2	3	4	5	N/A		

	Strongly Disagree	Disagree	Neither agree or disagree	Agree	Strongly Agree	Not Applicable	I prefer not to answer	Additional Comments
17. It was clear who was in charge of the staff	1	2	3	4	5	N/A		
18. Sometimes there was no-one available to deal with aspects of my care	1	2	3	4	5	N/A		
19. On at least one occasion a member of staff was not able to carry out a task that they should have been able to do	1	2	3	4	5	N/A		
The following aspects of the ward made it uncomfortable for me:								
20. Noise levels	1	2	3	4	5	N/A		
21. Poor lighting	1	2	3	4	5	N/A		
22. Temperature	1	2	3	4	5	N/A		
23. Poor cleanliness	1	2	3	4	5	N/A		
24. Lack of space	1	2	3	4	5	N/A		
25. Other - Please specify								
26. I felt that the attitude of staff towards me was poor	1	2	3	4	5	N/A		
27. It wasn't clear to me who was in charge of my care	1	2	3	4	5	N/A		
28. Staff always seemed to know what they were meant to be doing	1	2	3	4	5	N/A		

	Strongly Disagree	Disagree	Neither agree or disagree	Agree	Strongly Agree	Not Applicable	I prefer not to answer	Additional Comments
29. There were enough staff on the ward to get things done on time	1	2	3	4	5	N/A		
30. Staff gave me different information about my care	1	2	3	4	5	N/A		
31. Staff/patients waited a long time for porters to arrive	1	2	3	4	5	N/A		
32. Staff did not work together as a team here	1	2	3	4	5	N/A		
33. There was equipment that staff found difficult to use (e.g. monitoring equipment, beds, hoists)	1	2	3	4	5	N/A		
-Please specify any examples:								
34. I have needed treatment and there has been no-one available who was qualified to do it	1	2	3	4	5	N/A		
35. Staff were kept waiting for my test results	1	2	3	4	5	N/A		

	Strongly Disagree	Disagree	Neither agree or disagree	Agree	Strongly Agree	Not Applicable	I prefer not to answer	Additional Comments
36. Nurses were always able to get help from other staff when they asked for it	1	2	3	4	5	N/A		
37. Equipment needed for my care was always working properly	1	2	3	4	5	N/A		
-Please specify any problems								
38. Staff seemed to struggle to get help when they needed it	1	2	3	4	5	N/A		
39. Equipment and supplies were not always available when needed (e.g. hoists, bed pans, drugs)	1	2	3	4	5	N/A		
-Please specify any problems								
40. Staff always agreed about my treatment/care	1	2	3	4	5	N/A		
41. I always felt staff listened to me about my concerns	1	2	3	4	5	N/A		
42. Doctors were always able to get help from other staff when they asked for it	1	2	3	4	5	N/A		

	Strongly Disagree	Disagree	Neither agree or disagree	Agree	Strongly Agree	Not Applicable	I prefer not to answer	Additional Comments
43. When staff talked about my care with others the information they shared was correct	1	2	3	4	5	N/A		
44. Information about me that my health care team needed was always available	1	2	3	4	5	N/A		

ii) PATIENT-REPORTED INCIDENT TOOL (PIRT)

1) Date of report: ___________

2) We would like to know about the things that may have concerned you about your care, or the care of others, during this hospital stay. Please tell us what happened with your concern or experience, in as much detail as you can.

3) Why do you feel this was a safety concern for you?

4) What do you think could be done to stop this from happening again to you or other patients, in the future?

5) On a scale of 1-10 how serious do you think your safety concern was?

1 2 3 4 5 6 7 8 9 10

Not serious at all — Extremely serious

6) Do you think it would have been possible to have stopped your experience from happening?

Definitely yes	Probably yes	Probably not	Definitely not	Don't know
☐	☐	☐	☐	☐
