# Supplementary material for: The patient reporting and action for a safe environment (PRASE) intervention: a feasibility study
Source: BMC Health Serv Res. 2016 Nov 28;16:676. doi: 10.1186/s12913-016-1919-z (PMC5127050; doi:10.1186/s12913-016-1919-z)
Supplement: Additional file 3: — Example PRASE feedback report. (PDF 312 kb) [file 12913_2016_1919_MOESM3_ESM.pdf]

Appendix 3

PRASE FEEDBACK REPORT: Ward X

This report represents the results from the data collection within Ward X, using the PRASE Intervention toolkit. This report provides information from two tools:
Patient Measure of Safety (PMOS)
Patient-led patient safety Incident Reporting Tool (PIRT)

These data were collected between xx.xx.xx and xx.xx.xx. The demographics for this data collection were as follows:

Total patients recruited	
Total questionnaires completed	
Total number patient reported safety concerns	
Total number patient reported positive experiences	

How to interpret this report

PMOS SAFETY QUESTIONNAIRE SUMMARY FEEDBACK

- The PMOS questionnaire gives wards information across 9 key domains as follows:
    - *Communication and teamwork:* Responsiveness to patient and team working
    - *Type and layout of ward:* the Patient's experience of the ward environment
    - *Organisation and care planning:* Factors related to the care plan, and the availability of resources for the care plan
    - *Staff training*: Access to correct, timely and appropriate training and support
    - *Staff roles and responsibilities*: Clear supervision and lines of accountability for staff
    - *Access to resources*: Availability of experienced staff, equipment and external resources
    - *Information flow*: Availability of information about the patient
    - *Equipment* (design and functioning)
    - *Delays*
- A summary of the scores for each of the domains is presented first, with each domain then further detailed, with responses to individual questions presented.
- All graphs are displayed using a traffic light system – dark red indicates the most negative response possible, dark green indicates the most positive response possible.
- The answers respondents gave are shown in percentages of the total valid response for each domain or question. For the purposes of the graph we have not included 'not applicable' options therefore the total number of responses for each question may vary
- In answering some questions, patients would want to provide more information about their response. This is presented in two ways:
    - Via a comment – this is simply to add more explanation about their response, or contextualise it.
    - Via a patient 'report' – this is where a patient felt that their response required detailing further as a more significant safety concern, or positive experience.
    - Where either a comment or a patient report have been linked to a particular question, this is shown in the feedback.

PATIENT REPORTS OF SAFETY CONCERNS OR POSITIVE EXPERIENCES OF CARE

- Having completed the PMOS questionnaire, patients were also asked if there was anything they would like to tell us about their care.
- This was collected in two ways:
  - things that may have concerned them about their care, or the care of others, during this hospital stay
  - the good experiences of care either they, or others, had during this hospital stay
- Where patients reported concerns about their care, they were asked to provide further information on:
  - What happened, and why this was a concern for them (from a patient perspective)
  - What might be done to prevent it happening again
  - A patient rating of preventability (from a patient perspective)
    - Response options: ‘definitely preventable’, ‘probably preventable’, ‘probably not preventable’, ‘definitely not preventable’
  - A patient rating of severity (from a patient perspective)
    - Response options: 1-10, where 1=not serious at all, through to 10=extremely serious
- Where patient reports (either positive or a safety concern) were not linked to any particular question in the PMOS questionnaire, they are shown at the end of the report.
- **Please note all patient comments, reported safety concerns or positive experiences of care are reported verbatim and in italics.**

Section 1: PMOS Scores and related comments and patient reports

OVERALL SAFETY PROFILE

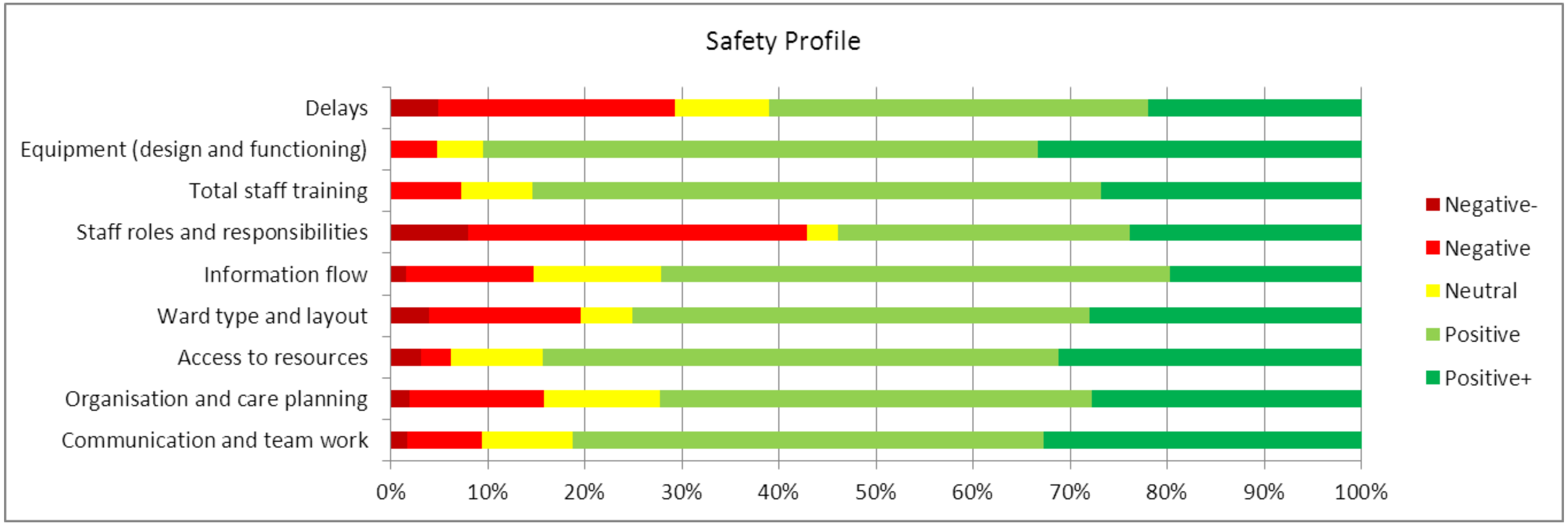

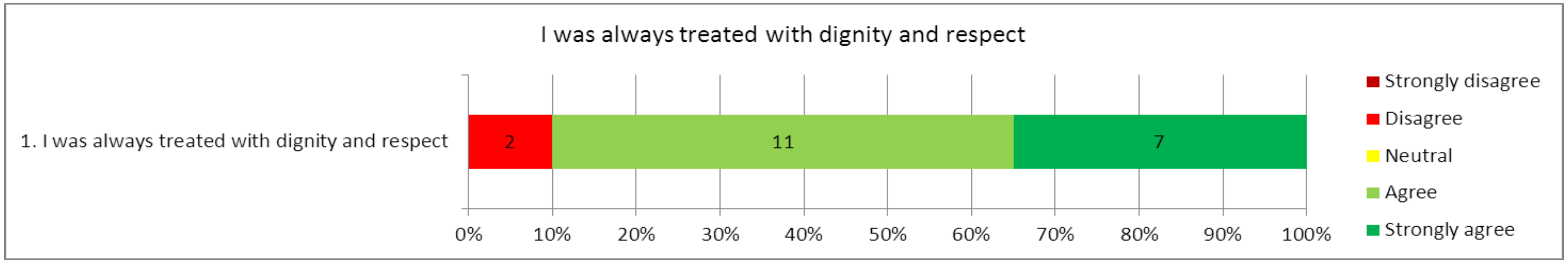

DOMAIN 1 – Communication and team-working

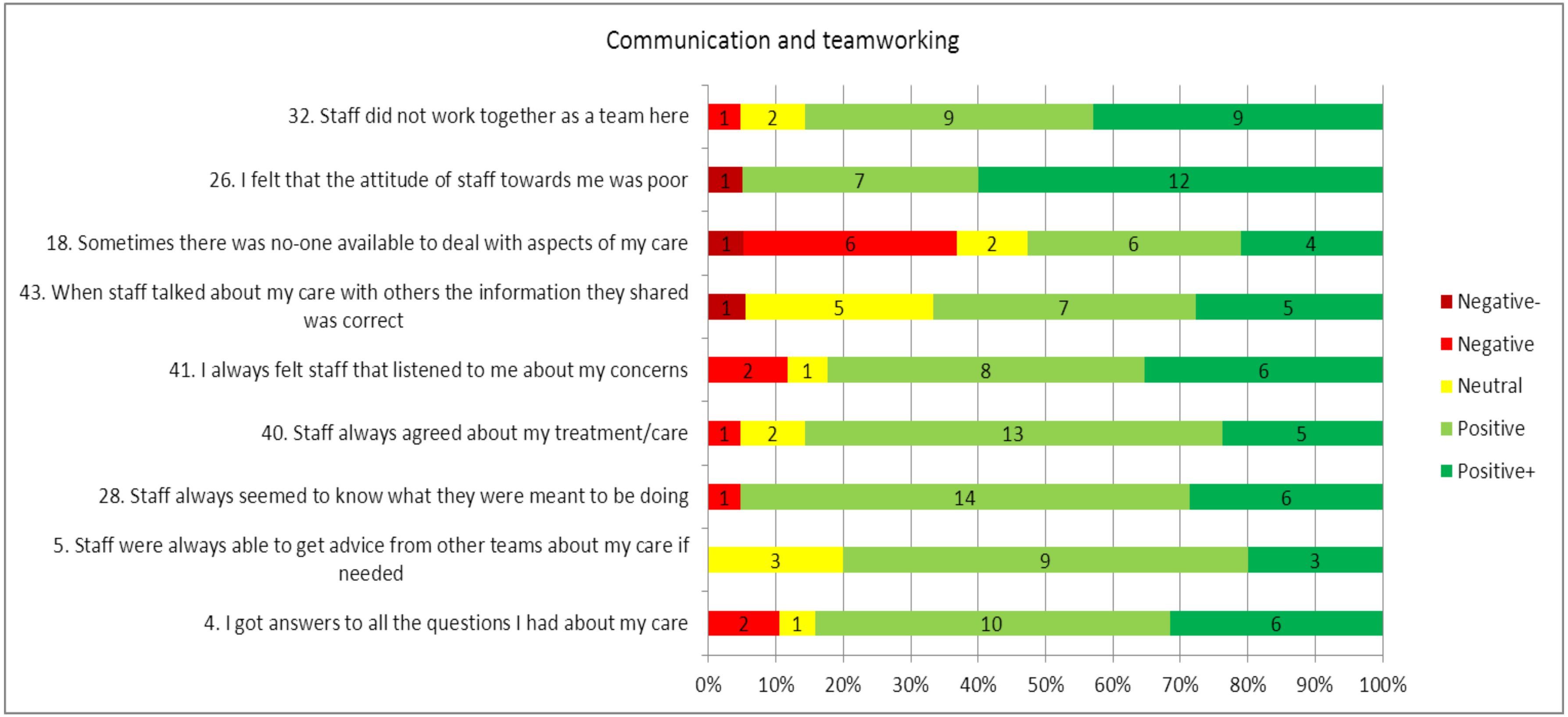

Patient comments linked to this domain:

Question No.	Question Text	Patient Comment
4	I got answers to all the questions I had about my care	*I did not know who to ask or they were not always available. I am still not sure what they have done to me. I would have liked to see the surgeon.*
5	Staff were always able to get advice from other teams about my care if needed	*Physiotherapists particularly good.*
		Information from GP about medication.
43	When staff talked about my care with others the information they shared was correct	*On my sheet my birthday has been wrong for a few days. It says the 21st but It's the 31st but they always agreed with me when I said the 31st.*
		No idea.
18	Sometimes there was no-one available to deal with aspects of my care	*Yes but not for long.*
		Sometimes, because the staff are busy.
		Not been in long enough.
		Not me but noticed adjacent patient on the commode for a long time. When you've had an accident and feeling unwell, it is something you don't need but impossible for staff to be on the spot for everyone.
		Only briefly.
26	I felt that the attitude of staff towards me was poor	*On a day shift their attitude is bad not just towards me but towards everyone. On the day shift I hate waking up in the morning.*
		Now and again just a little grumpy.

Patient reports of safety concerns linked to this domain:
None

Patient reports of positive experiences of care linked to this domain:

**Question No.**	**Question Text**	**What happened / why did it happen?**
26	I felt that the attitude of staff towards me was poor	*Very friendly and helpful. Cannot praise the staff enough.*
32	Staff did not work together as a team here	*They always work as a team here.*

Section 2: Patient reported safety concerns or positive experiences of care

A. Patient reported safety concerns not linked to a specific question

What happened / why did it happen?	What can be done to prevent it happening again?	Patient-rated preventability	Patient-rated severity
No washing facilities were offered in the morning, had to ask for them. Also no hand washing facilities were offered after patient had used the bedpan. Whilst making this report, the patient overheard the patient in the next bed also asking for help with washing. Do not feel clean, and want to wash and brush teeth. It is un-hygienic not to be able to wash hands after using bedpan.	*Offer wash bowls and help to all patients who need it. Do not wait for them to ask. Offer hand washing facilities after bedpan use.*	Definitely yes	7
Worried mainly about after-care. I will have to go to bed when the home care team come, not when convenient to me. I understand why this is the case but it's not great.	*I have been an engineer all my life and I am sure there must be a way of adapting a piece of equipment to help me pull up covers so I can do it myself.*	Probably yes	4
I feel that I have been treated like an 'object' very nicely; none of the medical team have told me what they have done to me or checked my wound or dressing, I don't know what my plan of care is. I didn't know there was someone in charge of the ward that I could talk to about my care. I have written a list of questions and am hoping for someone to talk to.			
Some food not good enough to eat - but not that hungry.			

B. Patient reported positive experiences of care

The following positive experiences of care were reported:

- Everything that has happened to me since I got here has been brilliant.
- Generally friendly attitude, especially the domestic staff.
- Generally the staff are so conscientious and always willing to help.
- Baby type mugs to hold hot tea - they cool them down
