# Supplementary material for: The patient reporting and action for a safe environment (PRASE) intervention: a feasibility study
Source: BMC Health Serv Res. 2016 Nov 28;16:676. doi: 10.1186/s12913-016-1919-z (PMC5127050; doi:10.1186/s12913-016-1919-z)
Supplement: Additional file 4: — Observation template for the Action Planning Group meetings. (PDF 75 kb) [file 12913_2016_1919_MOESM4_ESM.pdf]

Appendix 4

Observation template

Date of meeting	
Meeting number (e.g. first, second)	
In attendance	
Venue	
Start time	
End time	
Notes	

a) Notes on introduction given by PRASE team
b) Notes on introduction given by ward staff
c) How has feedback report been cascaded prior to meeting?
d) Responses from staff to report (specific issues/domains)
e) Feedback on format of report
f) Action Plan Process – written in meeting? - written post-meeting? Responsibility for?
g) General notes e.g. format of meeting / staff engagement etc

PLEASE OBTAIN COPY OF COMPLETED ACTION PLAN

Interview topic guide

Interviews will review the action plans set as part of the intervention, & discuss:

- General experience of the process (setting & implementing actions)
- Reflection on whether or not the action plans have been implemented
- Reasons given for these levels of implementation
- Support required by Ward teams to implement actions more successfully? (either from the research team, or from the hospital)

Researcher to input a summarised rating for level of implementation for each action on their plan (yes, no, partial).
